# Factors Affecting Impulse Buying Behavior of Consumers

**DOI:** 10.3389/fpsyg.2021.697080

**Published:** 2021-06-02

**Authors:** Rosa Isabel Rodrigues, Paula Lopes, Miguel Varela

**Affiliations:** Instituto Superior de Gestão, Lisbon, Portugal

**Keywords:** consumer behavior, purchase intention, impulse purchase, emotional influences, marketing strategies

In recent years, the study of consumer behavior has been marked by significant changes, mainly in decision-making process and consequently in the influences of purchase intention (Stankevich, 2017).

The markets are different and characterized by an increased competition, as well a constant innovation in products and services available and a greater number of companies in the same market. In this scenario it is essential to know the consumer well (Varadarajan, 2020). It is through the analysis of the factors that have a direct impact on consumer behavior that it is possible to innovate and meet their expectations. This research is essential for marketers to be able to improve their campaigns and reach the target audience more effectively (Ding et al., 2020).

Consumer behavior refers to the activities directly involved in obtaining products /services, so it includes the decision-making processes that precede and succeed these actions. Thus, it appears that the advertising message can cause a certain psychological influence that motivates individuals to desire and, consequently, buy a certain product/service (Wertenbroch et al., 2020).

Studies developed by Meena (2018) show that from a young age one begins to have a preference for one product/service over another, as we are confronted with various commercial stimuli that shape our choices. The sales promotion has become one of the most powerful tools to change the perception of buyers and has a significant impact on their purchase decision (Khan et al., 2019). Advertising has a great capacity to influence and persuade, and even the most innocuous, can cause changes in behavior that affect the consumer's purchase intention. Falebita et al. (2020) consider this influence predominantly positive, as shown by about 84.0% of the total number of articles reviewed in the study developed by these authors.

Kumar et al. (2020) add that psychological factors have a strong implication in the purchase decision, as we easily find people who, after having purchased a product/ service, wonder about the reason why they did it. It is essential to understand the mental triggers behind the purchase decision process, which is why consumer psychology is related to marketing strategies (Ding et al., 2020). It is not uncommon for the two areas to use the same models to explain consumer behavior and the reasons that trigger impulse purchases. Consumers are attracted by advertising and the messages it conveys, which is reflected in their behavior and purchase intentions (Varadarajan, 2020).

Impulse buying has been studied from several perspectives, namely: (i) rational processes; (ii) emotional resources; (iii) the cognitive currents arising from the theory of social judgment; (iv) persuasive communication; (v) and the effects of advertising on consumer behavior (Malter et al., 2020).

The causes of impulsive behavior are triggered by an irresistible force to buy and an inability to evaluate its consequences. Despite being aware of the negative effects of buying, there is an enormous desire to immediately satisfy your most pressing needs (Meena, 2018).

The importance of impulse buying in consumer behavior has been studied since the 1940's, since it represents between 40.0 and 80.0% of all purchases. This type of purchase obeys non-rational reasons that are characterized by the sudden appearance and the (in) satisfaction between the act of buying and the results obtained (Reisch and Zhao, 2017). Aragoncillo and Orús (2018) also refer that a considerable percentage of sales comes from purchases that are not planned and do not correspond to the intended products before entering the store.

According to Burton et al. (2018), impulse purchases occur when there is a sudden and strong emotional desire, which arises from a reactive behavior that is characterized by low cognitive control. This tendency to buy spontaneously and without reflection can be explained by the immediate gratification it provides to the buyer (Pradhan et al., 2018).

Impulsive shopping in addition to having an emotional content can be triggered by several factors, including: the store environment, life satisfaction, self-esteem, and the emotional state of the consumer at that time (Gogoi and Shillong, 2020). We believe that impulse purchases can be stimulated by an unexpected need, by a visual stimulus, a promotional campaign and/or by the decrease of the cognitive capacity to evaluate the advantages and disadvantages of that purchase.

The buying experience increasingly depends on the interaction between the person and the point of sale environment, but it is not just the atmosphere that stimulates the impulsive behavior of the consumer. The sensory and psychological factors associated with the type of products, the knowledge about them and brand loyalty, often end up overlapping the importance attributed to the physical environment (Platania et al., 2016).

The impulse buying causes an emotional lack of control generated by the conflict between the immediate reward and the negative consequences that the purchase can originate, which can trigger compulsive behaviors that can become chronic and pathological (Pandya and Pandya, 2020).

Sohn and Ko (2021), argue that although all impulse purchases can be considered as unplanned, not all unplanned purchases can be considered impulsive. Unplanned purchases can occur, simply because the consumer needs to purchase a product, but for whatever reason has not been placed on the shopping list in advance. This suggests that unplanned purchases are not necessarily accompanied by the urgent desire that generally characterizes impulse purchases.

The impulse purchases arise from sensory experiences (e.g., store atmosphere, product layout), so purchases made in physical stores tend to be more impulsive than purchases made online. This type of shopping results from the stimulation of the five senses and the internet does not have this capacity, so that online shopping can be less encouraging of impulse purchases than shopping in physical stores (Moreira et al., 2017).

Researches developed by Aragoncillo and Orús (2018) reveal that 40.0% of consumers spend more money than planned, in physical stores compared to 25.0% in online purchases. This situation can be explained by the fact that consumers must wait for the product to be delivered when they buy online and this time interval may make impulse purchases unfeasible.

Following the logic of Platania et al. (2017) we consider that impulse buying takes socially accepted behavior to the extreme, which makes it difficult to distinguish between normal consumption and pathological consumption. As such, we believe that compulsive buying behavior does not depend only on a single variable, but rather on a combination of sociodemographic, emotional, sensory, genetic, psychological, social, and cultural factors. Personality traits also have an important role in impulse buying. Impulsive buyers have low levels of self-esteem, high levels of anxiety, depression and negative mood and a strong tendency to develop obsessive-compulsive disorders. However, it appears that the degree of uncertainty derived from the pandemic that hit the world and the consequent economic crisis, seems to have changed people's behavior toward a more planned and informed consumption (Sheth, 2020).

## Author Contributions

All authors listed have made a substantial, direct and intellectual contribution to the work, and approved it for publication.

## Conflict of Interest

The authors declare that the research was conducted in the absence of any commercial or financial relationships that could be construed as a potential conflict of interest.
